# The Importance of Pelvic and Leg Length Assessment in the Setting of Postpartum Lower Back Pain

**DOI:** 10.7759/cureus.25489

**Published:** 2022-05-30

**Authors:** Leonid Tafler, Danielle Wilson, Paul Tafler

**Affiliations:** 1 Primary Care, Touro College of Osteopathic Medicine, New York, USA; 2 Medicine, Touro College of Osteopathic Medicine, New York, USA; 3 Biological Sciences, University at Buffalo, Oceanside, USA

**Keywords:** postpartum pain, gynaecology and obstetrics, manipulation under anesthesia, women’s health, postpartum, osteopathic manipulative treatment (omt), osteopathic manipulative medicine (omm), lower back pain (lbp)

## Abstract

One of the most common complaints during pregnancy is lower back pain. Women believe that this will disappear after they give birth, however, there are a significant number of women who suffer from persistent, unresolving pain that affects their daily lives. Very often, women will wrongfully blame the epidural anesthesia as the cause, however, there are physiologic and anatomic changes that occur. Patients often experience persistent pain when there is an absence of proper postpartum management. The lack of proper management can lead to unresolved pelvic rotation and dislocation, resulting in short leg syndrome. The common treatment for postpartum lower back pain includes various analgesics, physical therapy, and steroid injections. However, with a proper pelvic assessment, treatment with osteopathic manipulation alone, or reinforced with anesthesia, can be successful. Here, we present a case in which a patient presented with persistent lower back two years postpartum. She had been treated with common modalities prior to coming to our clinic and was subsequently found to have a short leg. We discuss the importance of a proper pelvic and leg length assessment in women who present with postpartum lower back pain, as well as how to both prevent and treat short leg syndrome in postpartum patients.

## Introduction

A common complaint after delivery is lower back pain, which can be due to a variety of causes. In this paper, we discuss the importance of keeping sacropelvic dysfunction on the differential diagnosis as well as the importance of remembering to perform a structural exam.

As a fetus grows within the mother’s uterus, the mother’s body goes through both physiologic and structural accommodations. This is meant to provide the fetus with an optimal environment to grow. During pregnancy, the increased synthesis of hormones, such as relaxin and progesterone, results in ligamentous laxity, allowing for structural changes. It is normal to develop a gradual increase in the thoracic kyphosis, forward pelvic tilt, and increased lumbar lordosis as a counterbalance to the tilted pelvis [1]. Musculoskeletal changes in the vertebrae, pelvis, and sacrum result in a strenuous muscle-controlled balance, as opposed to a ligamentous and disk-oriented balance that is normally occurring outside of pregnancy. Changes also occur within the abdominal muscles; they distend as the uterus grows and reduce the ability to counterbalance. As there are changes in the anterior-posterior curves, spinal segments that send sympathetic (T10-L2) and parasympathetic (S2-S4) innervation to the pelvic organs can become facilitated, resulting in a lower pain threshold [2].

During delivery, the hormones progesterone and relaxin both assist the ligamentous laxity. As labor progresses, the hip joint, pelvis, and abdominal muscles undergo maximum strain, causing stress on the back, pelvis, as well as sacroiliac joints. Being in the lithotomy position exacerbates these strains by not allowing gravity to assist [2]. The changes that occur in both pregnancy and during delivery can persist into the postpartum period as well, which can result in lower back pain. Severe pain is most commonly due to sacroiliac joint dysfunction [3]. In most women, postpartum lower back pain resolves within six months [4]. However, some patients who experienced lower back pain during pregnancy have persistent pain up to three years postpartum [5].

Today, the standard of care for lower back pain in postpartum patients includes anti-inflammatories, physical therapy, acupuncture, and supports or braces. If these fail, slow-releasing glucocorticoid injections are introduced [6]. Osteopathic manipulative medicine (OMM) is a very effective option for patients. OMM is characterized by palpation skills that are used to diagnose neuromuscular abnormalities and techniques that are used to treat the diagnoses. The goals of OMM are to relieve pain, restore function, improve movement, enhance circulation, normalize nerve stimulation, and achieve homeostasis [2]. If the traditional osteopathic therapy failed, osteopathic manipulation treatment reinforced by anesthesia, also known as manipulation under anesthesia (MUA), is a technique of choice. Here, we describe a case of a postpartum patient with persistent lower back pain that did not respond to conservative therapy but was treated successfully with MUA.

## Case presentation

The patient is a 27-year-old P1G1 female, who developed lower back pain after being in the lithotomy position during labor with her first child two years prior. During labor, she received an epidural for pain management. The pain was radiating down to her left leg and was associated with numbness and tingling. She had been treated with anti-inflammatory medications, physical therapy, and steroid injections for two years, all with minimal relief. She received an MRI of the lumbar spine, which showed disc degeneration and facet arthropathy at L5-S1, large central and left-sided disc herniation compressing the thecal sac and proximal left S1 nerve root, and left posterior disc herniation at T12-L1 producing a mild epidural effect.

She presented to our clinic with a chief complaint of lower back pain, radiating to the left leg with numbness and tingling. She highly believed that her pain was due to the epidural anesthesia she received during delivery. A structural osteopathic exam revealed a posterior left ilium, tenderness over the left sacroiliac joint, and a positive sitting flexion test, which led to her diagnosis of short left leg syndrome with a 1-inch leg length discrepancy. She received a lumbar X-ray, which showed mild disc space narrowing at L3-L4 and L5-S1 levels, and no fracture or spondylolisthesis. She also received a pelvic X-ray, which showed mild to moderate arthropathy at the sacroiliac joints bilaterally with joint space narrowing and subchondral sclerosis. Osteopathic manipulation failed to be successful, and we treated her with MUA. We treated her focusing on her pelvic and sacral area, using typical osteopathic techniques, such as high-velocity low amplitude and myofascial release, both of which were specific to the patient's physical exam findings. MUA proved to be successful for her, she was pain-free, her leg length discrepancy had resolved, and there were no longer any significant osteopathic physical exam findings.

When pregnant again two years later, she returned to the clinic and reported that she continued to be pain-free.

## Discussion

Chronic lower back pain in postpartum women is one of the most common musculoskeletal complaints within this population. Most studies show that an estimated 50% of women will complain of lower back pain, regardless of what specifically causes this pain [7]. Sacroiliac joint dysfunction, which also falls under sacropelvic dysfunction, has a prevalence among postpartum patients of an estimated 89% of women who also had lower back pain during pregnancy. This means that an estimated 89% of women who had lower back pain during pregnancy were found to have sacropelvic misalignment. It should be noted that because these physical exam findings were present, it does not necessarily mean that that is what caused the patients' lower back pain but is a possibility. This study also showed that an estimated 70% of pregnant and postpartum women who underwent an osteopathic structural exam were found to have sacroiliac joint disorders. Structural exams are done using the sitting and standing flexion tests, where the examiner places their thumbs on the sacroiliac joints and the patient flexes their trunk forward and the examiner palpates which side they feel move first, the sacral rock test where the examiner has the patient lay prone and examines if the base of the sacrum is restricted when pressure is applied, and palpating the pelvis and sacral landmarks, such as the anterior superior iliac spines, the ischial tuberosities, and the posterior superior iliac spines, to properly diagnose a misalignment. Another part of the structural exam that should be performed is assessing the leg length by having the patient lay supine and measuring the discrepancy in where the two medial malleoli lay compared to one another. It is shown that leg length discrepancies greater than 2 cm can contribute to long-term pathology and lower back pain [8], however, in this case, the leg length discrepancy showed to be more of a marker of her sacropelvic dysfunction. Sacroiliac joint disorders affect the entire sacropelvic girdle, which can subsequently lead to short let syndrome [9]. Because lower back pain is so common, there are a variety of treatment options, such as physical therapy, stabilization belts, acupuncture, massage, nerve stimulation, muscle relaxants, pain relievers, relaxation, and yoga [7]. Due to the fact that there are many possible causes of postpartum lower back pain, these treatments don’t work for everyone, and many women walk around every day living with this chronic pain. Our patient tried many of these modalities, but they might not have worked because none of them fixed her core problem: musculoskeletal misalignment within the sacropelvic region, with subsequent short leg syndrome. Although she received radiographic imaging studies that showed some structural abnormalities, her previous failed trials of treatment with physical therapy, steroid injections, muscle relaxers, and pain relievers should have relieved her pain, however, she did not feel relief until she underwent OMM and MUA. Studies have shown that OMM significantly reduces pain and improves functionality in postpartum patients with lower back pain [10]. We described one case here, but in our practice, we have seen many postpartum patients with lower back pain who we diagnosed with short leg syndrome due to sacropelvic dysfunction, which previous physicians did not diagnose.

OMM is used to treat “somatic dysfunctions,” which are seen as responses within the musculoskeletal system to imbalance and improper function within the body. The purpose of OMM is to restore the improper function and imbalances within the body and achieve homeostasis. OMM can be done after a proper structural exam is performed. This includes looking for TART (texture changes, asymmetry, restricted range of motion, and tenderness) changes in order to obtain a proper diagnosis [2]. OMM under anesthesia has been used since the 1930s by orthopedic physicians and osteopathic physicians and has been used as an alternative to surgery or after surgical interventions have failed. The anesthetic drug commonly used for these procedures is propofol, which takes involuntary reflexes away and relaxes the muscles [11]. During the MUA, the patient continues to express pain with a face mimic, even though they are unconscious. This makes the procedure safe and avoids potentially harmful techniques done by the practicing physician. During the procedure, the barrier of resistance is met and articulatory motions to lyse fibrotic scar tissue are achieved and appear as resolved inflammation [12]. OMM under anesthesia for postpartum patients with lower back pain is a great treatment option. As the case with our patient, we were able to reach the proper barriers that her muscles and bones created, correct her musculoskeletal alignment, which she might not have tolerated while being awake, and her pain resolved.

**Figure 1:**
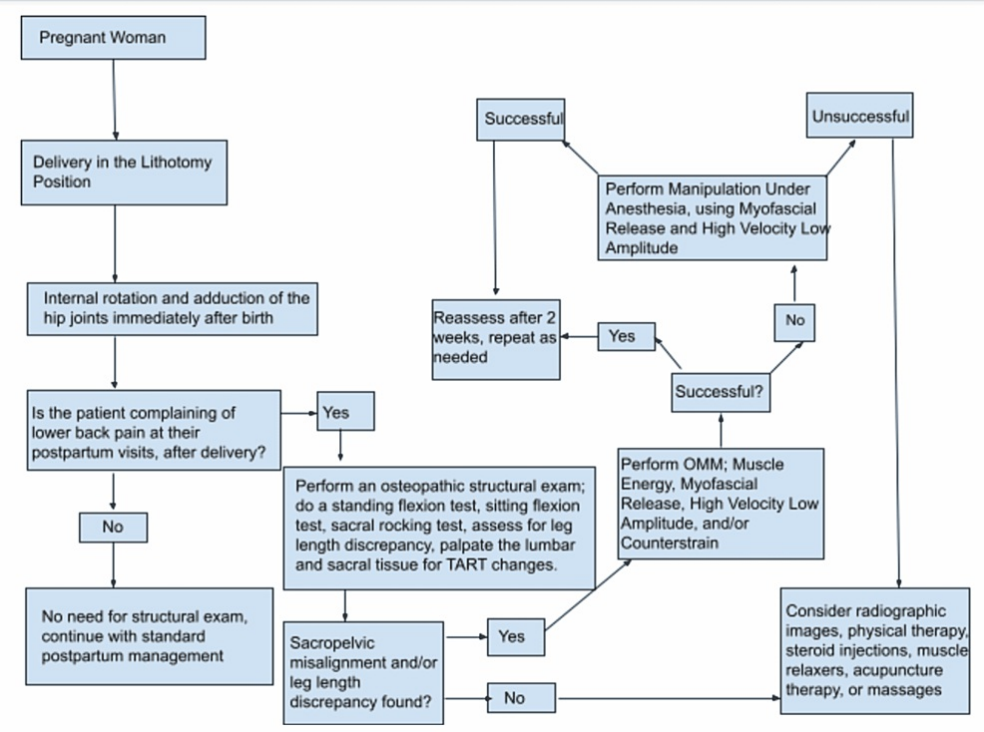
Postpartum Lower Back Pain Guideline Postpartum management refers to standard-of-care postpartum management done by obstetricians, which they learned within residency training. TART changes is a pneumonic used by osteopathic physicians to determine whether there is a musculoskeletal dysfunction or misalignment. TART: Tenderness to palpation, Asymmetry of the musculoskeletal alignment, Restricted range of motion, and Tissue texture changes, referring to erythematous or blanching skin, boggy or dry skin, or the muscles feeling rope-like or hypertonic OMT: Osteopathic Manipulative Medicine; MUA: Manipulating Under Anesthesia

Another issue that this case highlights are the lack of education on how to prevent lower back pain and musculoskeletal misalignment. As stated above, 50% of postpartum women experience lower back pain due to many causes, but that number could potentially decrease if there were proper education and management (Figure 1). According to an osteopathic approach to diagnosis and treatment, one way to prevent pathological pelvic changes and short leg syndrome is to internally rotate and adduct the hips from the lithotomy position, as you extend the hip joint so that the legs lay flat on the table. As mentioned earlier, the ligaments are more relaxed during pregnancy, birth, and the immediate postpartum phase due to the relaxin and progesterone released. This technique will place the hip in the proper position to reduce the risk of such musculoskeletal misalignment [2]. We would like to emphasize the importance of proper leg placement when the legs are coming down from the lithotomy position.

## Conclusions

An estimated 50% of patients will develop lower back pain due to a number of possible causes. Although we do not know the true incidence yet, physicians should keep sacropelvic pain high up on their differential diagnosis when postpartum patients present with a chief complaint of lower back pain. Implementation of teaching proper leg placement after birth during Obstetrics and Gynecology residency should be considered to hopefully prevent pain in some of these patients. The emphasis of this case report should be placed on increasing awareness of sacropelvic dysfunction potentially causing lower back pain in postpartum patients. We emphasize the importance of when a patient may need a proper structural exam, with specific consideration to the lumbar spine, sacrum, and pelvis, when they present with a chief complaint of lower back pain. It is also not typically taught that OMM under anesthesia is a viable treatment option. If physicians become more aware of this treatment option, patients would be more likely to receive OMM or MUA as opposed to, or in addition to, physical therapy, acupuncture, massages, etc. Overall, a physician should keep sacropelvic dysfunction high up on their differential list when seeing patients with postpartum lower back pain and should understand when a proper osteopathic structural exam may be needed. The incidence of lower back pain caused by sacropelvic dysfunction has not been researched, and we recommend that a research study be performed in the future to get the true incidence of lower back pain caused by sacropelvic dysfunction.
